# Conforming collections: assessing medical and allied health collections using Doody's Core Titles

**DOI:** 10.5195/jmla.2022.1114

**Published:** 2022-07-01

**Authors:** Efren Torres, Zipporah Dery, Raquel Samar, Marlon Gado

**Affiliations:** 1 emtorres@dlshsi.edu.ph, Special Assistant to the Vice Chancellor for Academics, De La Salle Medical and Health Sciences Institute, Dasmariñas City, Philippines; 2 zmdery@dlshsi.edu.ph, Director, Romeo P. Ariniego, MD, AFSC, Library, De La Salle Medical and Health Sciences Institute, Dasmariñas City, Philippines; 3 rpsamar@dlshsi.edu.ph, Chair, Collections Development and Access, Romeo P. Ariniego, MD, AFSC, Library, De La Salle Medical and Health Sciences Institute, Dasmariñas City, Philippines; 4 mggado@dlshsi.edu.ph, Collections Development Librarian, Romeo P. Ariniego, MD, AFSC, Library, De La Salle Medical and Health Sciences Institute, Dasmariñas City, Philippines

**Keywords:** collection development, medical and allied health collection, match, trend, Doody's Core Titles

## Abstract

This study assessed the print collection of an Asian academic medical library using list-checking. The library's book collection was matched to Doody's Core Titles (DCT) subspecialties to identify strong and weak subject areas and understand temporal trends from 2014 to 2020. Basic sciences and nursing were the strongest subspecialties from 2018 to 2020, with many subjects having 100% matches, likely because most academic programs share the same basic sciences foundation subjects and nursing collections had been developed for many years as a long-standing program of the institution. Associated health-related disciplines was the weakest subspecialty. These subjects need to be prioritized in collection development. All subspecialties exhibited an increasing trend of matching between 2014 and 2020. Electronic books were included in the matching to DCT 2020; however, the match was low compared to print only or both print and electronic titles. DCT title matching can not only identify gaps in library collections that need to be filled but also point toward opportunities to develop strong and varied collections in medicine and allied health.

## INTRODUCTION

Collection assessment is an important process to keep library collections relevant, authoritative, and updated. It is essential in the effective management of library resources and ensuring that libraries abide by their goals, users have access to the information that they need, and budget is appropriately allocated. Collection assessment is used for various purposes, such as planning and budgeting, accreditation, and monitoring of accountability, and enables libraries to determine the strengths and weaknesses of the collection using statistical data and personal judgment based on knowledge and experience. Collection assessment is an opportunity to develop collections with objectivity and soundness because it identifies areas of the collections that need to be maintained or improved [1].

Different types of collection assessment techniques are used. List-checking is among the qualitative assessment methods, which involves determining the percentage of titles included in the library collection against titles found on a list, such as a catalog, bibliography, subject compilation, list prepared by a professional organization or government body, or course syllabus. Advantages of list-checking include the availability of credible and current lists for use by libraries, the opportunity for librarians to become more familiar with resources for a specific subject, and the combination of qualitative and quantitative assessment brought by the judgment of the persons or organizations who compiled the list and the number of titles held in the library. However, this method poses disadvantages, such as potential bias of the compiler, inappropriateness of lists to meet the library's mission, and the persistence of out-of-date lists [1]. List-checking is used to gauge the strengths and weaknesses of library holdings and determine local user needs [2]. Although it is not the only method of assessing collections and determining quality, list-checking is a reliable and practical method of identifying the condition of collections.

### Review of related literature

Several previous studies have employed list-checking to assess collections on varied subjects. A toxicology collection was checked against a subject bibliography, and the strong and weak subjects were presented based on the percentage of titles found in the subject bibliography that were owned by the library. A library collection was evaluated against the references cited in the theses and dissertations of graduate students to determine the usefulness of the library collection to graduate students. Print and electronic cited references were matched against the Online Public Access Catalog (OPAC), which showed that 87% of the works cited were owned by the library and that there was an increase in library ownership of cited resources in education and social sciences but a decrease in the arts and humanities and sciences. Thirty-one percent of references were available electronically, and most electronic references cited were in the social sciences. A checklist developed by William F. Meehan III was used to assess the rowing collections of 2 university libraries, where titles were listed as exact match, near match, or no match, and strength and weakness of the collection were identified based on the matching. The football collection of university libraries was assessed using a core collection for football, and results showed that most libraries owned at least 50% of the titles in the list [2–5]. The collections of United States libraries were checked to determine the extent of holdings “that includes characters from racial and ethnic minorities, characters with disabilities, and characters who identify as LGBTQ” by developing 4 checklists and matching titles in the lists against the holdings of libraries [6]. Public libraries were observed to have more titles in the checklists, and several libraries did not own any titles in the checklists. Overall, these studies used library catalogs to identify titles owned by the libraries; categorized the titles as match, near match, and non-match; and determined the strength and weakness of the collection or subject based on the matching.

Several previous studies have also developed core lists to aid in selection and collection assessment, such as a nursing journal core list to assist nurses and librarians in journal evaluation and authors in selection for journal publication, and a core and essential monograph list for veterinary medicine based on the number of occurrences of titles in citation data, reading lists, textbook and reserve book lists, and published bibliographies. Core lists and list-checking are relevant methods for collection selection and assessment among libraries and provide guidance in building and maintaining quality and responsive collections based on criteria recognized as authoritative and relevant by the academe, profession, or industry [7–8].

### Doody's Core Titles

Doody's Core Titles (DCT) is an annual list of core titles for medicine and allied health published by Doody Enterprises, developed as a result of the discontinuation of the Brandon/Hill Selected List of medical, nursing, and allied health books that had long been used as a collection development guide by medical libraries [9]. DCT contains recommended core titles in 121 health science specialties in clinical medicine, basic sciences, nursing, allied health, and other associated health-related disciplines selected by content specialists and librarians based on 5 collection development criteria. An Essential Purchase Titles List is provided to help small libraries decide on which titles to buy out of the core titles list [10].

In the evaluation of DCT 2004, some flaws were noted in terms of the objectivity of the selection, but it was commended for the selection and rating criteria and deemed as an “important resource for health sciences librarians who are responsible for developing and maintaining monographic collections” [9]. Although DCT may be subjective due to the judgment brought by the selectors, it compensates for this weakness through its distinctive approach of pooling a “community of experts” composed of librarians, health professionals, subject experts, and technical staff to identify the best medical and allied health titles; thus, the selection decision is not based solely on a small group of individuals but rather on a collective assessment of a community. DCT “can assist collection development, aid collection assessment, serve as a recommended source for textbook selection, and provide an entry point into the literature of an unfamiliar discipline” [11]. It was used as a benchmark in developing an electronic reference collection, comparable to earlier studies that used DCT as a point of reference in collection assessment [12].

Several Philippine libraries utilize DCT in selection, or are familiar with it as a selection tool. In matching the collections of five libraries to DCT, two had a high percentage of match titles, two had a high percentage of near-match titles, and one had a low percentage of match titles. Library budget and the role of the library director in selection contributed to the likelihood of matched titles in the collection. Beyond this study, however, little research has been done on the use of DCT among Philippine medical libraries [13].

### Matching of collections of an academic library

The objective of this study was to assess the print collection of an academic library in the Philippines using the full DCT list. Specifically, it sought to identify the match of the print collection to DCT 2018, 2019, and 2020, determine the strong and weak subject areas based on the matching, and assess temporal trends in matching from 2014 to 2020. It focused on analyzing the print collection of one library based on the full DCT list rather than only the Essential Purchase Titles List.

The academic library is part of a tertiary educational institution that offers senior high school in STEM (science, technology, engineering, and mathematics) Track blended with health-related courses, undergraduate programs in nursing, physical therapy, occupational therapy, speech language pathology, medical radiation technology, nuclear medicine technology, medical laboratory science, pharmacy, and biochemistry; masters programs in nursing and public health; and a doctor in medicine program. It has a teaching hospital where undergraduate and doctor of medicine students perform internships. The library provides relevant and updated medicine and allied health resources to support the learning, teaching, and research of students, faculty members, non-teaching staff, and hospital staff such as residents, consultants, and fellows. It holds around 26,000 titles of print books with an annual acquisition rate of 1,500 to 2,000 print volumes from 2014 to 2020. It uses DCT as a collection assessment guide to keep its collections responsive to the needs of its users and the medical and allied health professions.

List-checking is utilized in collection assessment for this library because it is a straightforward way to determine strengths, weaknesses, and gaps and can be performed by a lean team of librarians. The school administration tends to approve collection acquisitions based on benchmarks, such as bibliographies and core lists, and DCT is used among the benchmarks for the collection. List-checking is also utilized to comply with the accreditation requirements set by the local regulatory body in the country. The national accreditation looks into several aspects of the library, including the quantity, quality, format, content, and age or publication year of the collection. Collection assessment and evaluation against standard bibliographies and lists are performed, and resultant reports and documentation are submitted as part of the library's collection management during accreditations to serve as evidence of the quality of the collection.

DCT was chosen as an assessment tool because of its subject specialization and strengths as a selection tool for medicine and allied health sciences. Its covers five subspecialties that correspond to a range of allied health professions, the basic sciences, and medical specialties. The range of subjects under the subspecialties complement the medicine and undergraduate programs offered by the institution. A separate subspecialty for nursing consists of subjects spanning basic and advanced nursing subjects and complements the bachelors and masters programs in nursing offered by the institute. Medicine education is a flagship program of the institution, offering a doctor of medicine program and residency programs for many specializations. The comprehensive list of basic sciences and clinical medicine subspecialties complements the subjects in the doctor of medicine program and residency programs, which support the information needs of medical students, residents, and practicing physicians at varying levels.

The library has been performing annual collection assessment using DCT from 2014 to present. Other lists in evaluating collections are also used, such as national guidelines for the undergraduate and graduate courses offered in higher education that list recommended reference books. These guidelines set minimum standards, and the library ensures that the titles listed in the guidelines are available in its holdings. However, not all programs have a list of recommended titles; only the minimum number of books in the collection is given. For programs with a recommended list, not all titles listed in the guidelines are included in the DCT list, and not much information is available on the criteria used to select the titles listed in the guidelines except that the titles are required by the national accrediting bodies. The library also collects local literature in medicine and allied health sciences, although there are not many local authors who publish books in these subjects, and there is no core list developed specifically for local literature in these subjects. The library strives to consider these lists in collection building along with DCT.

The study can be helpful to administrators, librarians, faculty members, and students because it can be used as a basis to develop high-quality medicine and allied health collections according to an industry-accepted standard. Gaps in the collection can be identified and improved, whereas strong collections in specific subjects can be maintained.

## METHODS

In this descriptive study, we used list-checking to assess the print collection of the library from 2014 to 2020 based on the full list of DCT 2014 to DCT 2020. DCT has five subspecialties: associated health professions, basic sciences, clinical medicine, associated health-related disciplines, and nursing. Titles were exported from the DCT website (http://www.doody.com/dct/) for each year to Microsoft Excel and arranged by subspecialty, then manually searched in the library's online public access catalog. The library uses Destiny Library System in organizing and managing its collections. All titles owned by the library and encoded in the catalog were included in the search. Titles with the same edition found in the catalog were marked as exact matches. Titles with a superseded edition found in the catalog were marked as near matches. Titles not found in the catalog were marked as non-matches. Previous studies employed that same methodology. The collections development librarian was tasked to export, search, and compare titles. Data were reviewed and verified by the head librarian to ensure accuracy [3–5].

Match titles per subspecialty were computed by combining exact and near match titles and calculating its percentage. Strong and weak areas were identified based on the percentage of matching. Temporal trends were determined by comparing the percentage of matching in subspecialties from 2014 to 2020.

DCT subjects dental auxiliaries, dentistry, optometry, and veterinary medicine were excluded from the matching because corresponding programs were not offered by the institution. Collection development is based on the programs offered, and the library does not prioritize the acquisition of titles for subjects that have no program complement, which would result to no or low matching to DCT titles.

## RESULTS

### Match of the print collection based on DCT subspecialties

Out of 117 subjects covering 5 subspecialties in DCT 2018, 21 had a 100% match (Table 1). Most matches were in the basic sciences, which complemented the curriculum of most courses offered. Many subjects had a 50–75% match, with the majority in nursing and clinical medicine, and 9 subjects had <50% match, most of which were associated health-related disciplines subjects.

**Table 1:** Match percentage of subspecialties to DCT 2018

Match percentage	Subspecialties, number of titles					Total	%
	Associated health professions	Basic sciences	Clinical medicine	Associated health-related disciplines	Nursing		
100%	3	9	3	0	6	21	17.95 %
76%–99%	7	3	21	0	4	35	29.91 %
50%–75%	3	0	22	3	24	52	44.44 %
<50%	0	0	3	6	0	9	7.69%
Total	13	12	49	9	34	117	100.00 %

The percentage of subjects with 100% match decreased while the percentage of subjects with 76%–99% match slightly increased in 2019 (Table 2). Most of the subjects had 50%–75% match, similar in 2018. However, the percentage of subjects with less than 50% match increased in 2019. Most of the Basic Sciences titles were 76%–99% match while the majority of the titles under this subspecialty were 100% match in 2018. Clinical Medicine titles were in 76%–99% and 50%–75% match brackets while a number of the Nursing titles were in 50%–75% match, comparable in 2018.

**Table 2:** Percentage of match of the subspecialties to DCT 2019

Match percentage	Subspecialties, number of titles					Total	%
	Associated health professions	Basic sciences	Clinical medicine	Associated health-related disciplines	Nursing		
100%	3	4	2		5	14	12.17 %
76%–99%	4	7	16		8	35	30.43 %
50%–75%	4	1	16	1	18	40	34.78 %
<50%			15	8	3	26	22.61 %
Total	11	12	49	9	34	115	100%

The shift to full online learning in 2020 due to the COVID-19 pandemic prompted the library to realign its budget to purchase or subscribe to more electronic books. The decline in the percentage of 100% match in several subjects in 2019 caused a concern for the library, and the subscription to more electronic books in 2020 led to the inclusion of electronic books in the 2020 collection assessment. We were also interested to know if electronic titles had an impact on the percentage of match. Print books were only considered in the previous DCT assessments, and the acquisition of more electronic books to support online learning in 2020 was among the changes made in that year's acquisition plans.

Instead of presenting the match per subject under each subspecialty, the match per title under each subspecialty was provided to check the impact of electronic titles in the matching (Table 3). Basic Sciences titles garnered the highest number of matches while Associated Health-related Disciplines had the lowest number of matches. Clinical Medicine and Nursing subspecialties retained high percentages of match, similar to the previous years. Most of the match titles across the subspecialties were in print and both in print and electronic. Electronic-only titles accounted for a small number of matches.

**Table 3:** Percentage of match of the subspecialties to DCT 2020

Subspecialty	Total number of DCT titles	Number of match titles	Percentage of match titles	Number of match titles (Print only)	Number of match titles (Print and Electronic)	Number of match titles (Electronic only)
Associated Health Professions	453	293	65%	250	26	17
Basic Sciences	183	169	92%	106	58	5
Clinical Medicine	1,148	823	72%	484	273	66
Nursing	540	405	75%	350	48	7
Associated Health-related Disciplines	145	54	37%	51	2	1
Total	2,469	1,744	71%	1,241	407	96

### Strong and weak subject areas based on DCT subspecialties

Basic sciences and nursing were the strongest subspecialties, for which many subjects were 100% match for 3 years (Table 4). This tendency for high matching is likely because most academic programs at the institution share the same basic sciences foundation subjects. The nursing program has been offered for many years, which allowed the library to develop its collections longer than the other courses. Strong subjects matching the undergraduate programs offered by the academic institution were pharmacy, speech, language and hearing, radiologic technology, and laboratory technology, among the associated health professions. Medical Laboratory Science, Pharmacy, and Speech Language Pathology were new programs offered by the institute, which could explain the tendency to provide collection support to programs offered. Dictionaries/terminology was consistently the strongest subject under associated health-related disciplines.

**Table 4:** Strong areas per subspecialty Subsp ecialty

Subspecialty	Subjects with the highest match (i.e., strongest subjects)		
	2018	2019	2020
Associated Health Professions	Pharmacy (100%) Radiologic Technology (100%) Speech, Language, and Hearing (100%)	Radiologic Technology (100%) Medical Physics (100%) Laboratory Technology (100%)	Laboratory Technology (100%)
Basic Sciences	Anatomy/embryology (100%) Biochemistry (100%) Immunology (100%) Microbiology (100%) Molecular biology (100%) Neuroscience (100%) Pathology (100%) Pharmacology (100%) Physiology (100%)	Biochemistry (100%) Molecular Biology (100%) Neuroscience (100%) Pathology (100%)	Anatomy/emb ryology (100%) Pathology (100%) Pharmacology (100%)
Clinical Medicine	Diagnostic Radiology (100%) Radiation oncology (100%) Surgical Pathology (100%)	Laboratory Medicine (100%) Rheumatology (100%)	Pediatrics (100%) Radiation oncology (100%)
Associated Health-related Disciplines	Dictionaries/Terminology (67%)	Dictionaries/Terminology (67%)	Dictionaries/Terminology (64%)
Nursi ng	Administration/Management (100%) Diagnosis/asse ssment (100%) Fundamentals (100%) Laboratory (100%) Nursing process (100%) Research (100%)	Administration/Management (100%) Informatics (100%) Perinatal (100%) Education (100%) Nursing Research (100%)	Administration/Management (100%) Diagnosis/Ass essment (100%) Laboratory (100%) Nursing process (100%)

Similar subjects were observed to be the weakest subjects across three years, such as clinical psychology, epidemiology, plastic and reconstructive surgery, managed care, and home care (Table 5). Associated health-related disciplines was the weakest subspecialty having low match, perhaps because these subjects are not taught in many of courses offered, meaning that books on these subjects may not be given as much attention as other subjects that are shared by several courses. Clinical psychology is not offered as a course, resulting in a low match. History of medicine had no match in 2018, which was surprising given that this is considered a foundation topic, but this improved in 2019 and 2020.

**Table 5:** Weak areas per subspecialty

Subspecialty	Subjects with the lowest match (i.e., weakest subjects)		
	2018	2019	2020
Associated Health Professions	Psychology, Clinical (56%)	Psychology, Clinical (56%)	Emergency Medical Services (58%) Psychology, Clinical (58%)
Basic Sciences	Epidemiology (92%)	Epidemiology (62%)	Epidemiology (78%)
Clinical Medicine	Oncologic Surgery (35%)	Plastic & Reconstructive Surgery (24%)	Plastic & Reconstructive Surgery (23%)
Associated Health-related Disciplines	History of Medicine (0%)	Managed Care (14%)	Managed Care (13%)
Nursing	Ambulatory (50%)	Home Care (40%) Oncology (40%)	Home Care (40%)

### Temporal trend in matching from 2014 to 2020

The library owned an average of 22,660 titles from 2014 to 2020. Over this time period, the total percentage of matching titles was observed to increase starting in 2015, which is when the library began acquiring books based on DCT (Table 6). The budget allocated to print books also increased over this time period but dipped in 2020 when the pandemic hit and budget had to be realigned to subscribe to more electronic resources.

**Table 6:** Number of titles owned by the library and percentage of DCT matching titles from 2014 to 2020

Year	Library budget for print collection (USD)	Number of titles held by the library	Number of titles in DCT	Number of DCT match titles (percentage)
2014	190,000	19,593	2,424	1,004 (41%)
2015	230,000	19,951	2,383	1,562 (66%)
2016	230,000	22,374	2,428	1,441 (59%)
2017	249,000	24,027	2,382	1,503 (63%)
2018	249,000	22,778	2,266	1,667 (74%)
2019	215,999	23,941	2,211	1,485 (67%)
2020	108,000	25,953	2,469	1,744 (71%)

**Figure 1:**
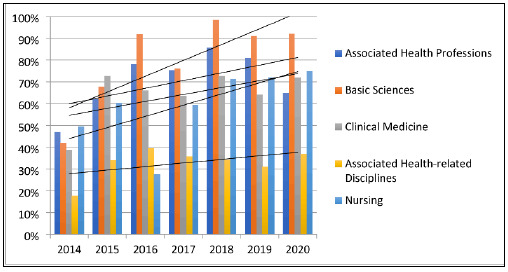
Temporal trends in DCT matching titles per subspecialty from 2014 to 2020

All subspecialties exhibited increasing trends in matching based on DCT 2014 to DCT 2020 (Figure 1). However, some subjects had no or low matching, and the associated health-related disciplines subspecialty was the weakest in terms of percentage of match. These trends demonstrate that action is taken to respond to the gaps in the collection through conscious selection each year aided by the previous year's collection assessment. However, the total percentage of match for associated health professions dipped in the last three years, requiring attention from the library considering that several subjects under this subspecialty correspond to new programs offered.

## DISCUSSION

Subspecialties with common subjects taught across different academic programs, such as the basic sciences, tended to have a high percentage of DCT matching titles. Subspecialties associated with long-standing programs offered by the institution, such as medicine and nursing, also tended to have a high rate of matching because the library was able to develop the collections for these subjects longer than the other allied health programs that have been recently offered.

Acquisition of titles is dictated by the curriculum support needed for the programs offered by the institution. This was evident in the subjects of pharmacy; radiologic technology; speech, language, and hearing; biochemistry; and medical laboratory science. There is an opportunity to continue developing the collections for existing programs and to start building collections based on new programs to be offered in the future. Conversely, titles corresponding to programs that are not offered are unlikely to be acquired because there is no curriculum to support and associated budget.

Some subjects had no or a low percentage of DCT matching titles. Associated health-related disciplines remained the weakest subspecialty across years. Although some of these subjects are taught in a smaller number of courses than other subjects and may be regarded as minor subjects, collections in these areas need to be developed to create well-rounded and balanced holdings of both major and minor subjects. Strong and weak subjects were consistent in some subspecialties, such as basic sciences and nursing, but changed over time in clinical medicine subspecialties. As the curriculum of these programs did not change in recent years, the shift in subjects with high and low percentages of matches indicates changes in selection priorities or unavailability of titles.

Concerning the unavailability of book titles, the library performs regular book selections locally and internationally, and an annual budget is allocated for the acquisition of book titles. Most books are sourced abroad due to lower costs offered by international publishers and distributors compared with local distribution channels. However, there are instances where some titles are not available regionally from Asian book suppliers and publishers due to costs and restrictions in publication and shipments. Shipping costs and duration of shipment discourage the library from purchasing books outside of Asia. This was a challenge for us in 2020, when the annual book selection had to be done on campus using catalog lists supplied by international publishers and budgets for print books were cut. There was a decrease in the percentage of match of associated health professions, and there were less subjects with 100% match in 2020 compared with the previous years.

Electronic titles are purchased or subscribed to in order to complement the print collection. National accrediting bodies usually prescribe the acquisition of print books instead of electronic formats, such that the print collection is usually the benchmark of library collections. However, electronic formats are now slowly being recognized alongside print collections, giving more reasons for local libraries to build electronic collections. The transition to online and remote learning brought about by the COVID-19 pandemic also prompted libraries to start building or continue maintaining electronic book collections. Our library has been buying or subscribing to electronic books for several years, usually in the form of database packages or collections because these are cheaper and more cost-efficient. The high cost of the pick-and-choose model of individual electronic book titles is prohibitive and not usually an option chosen by our library. Similar to the purchase of print books by title, the acquisition of electronic books may affect the percentage of matching if it is also done by title. As shown in the 2020 assessment, the number of electronic books matched was low compared to print only or both print and electronic. Packages and collections do not offer much flexibility in choosing titles included in the bundle, so it could not be guaranteed if these would include DCT-listed titles. Selection of books by title allows the library to choose specific titles based on need, such as compliance to DCT. This shows that title-based selection of books, whether print or online, should be considered to ensure that library collections comply with DCT.

School administrators and faculty members at our institution actively participate in book selection. However, not all are aware of DCT and may rely on their own preference instead of using DCT as a guide during selection. Recommended titles listed in national guidelines must be included in the collections but may not be listed in DCT. Books with local relevance may also not be included in DCT. Priority given to books listed in national guidelines and locally published or contextualized titles may also affect the selection of DCT-listed titles. Libraries need to address deficiencies in these subjects to close gaps in the collection by working closely with faculty members to encourage them to consider DCT titles in the selection, aside from choosing titles based on faculty preference, recommended lists from the national guidelines, and local titles. This is an opportunity for libraries to connect with faculty members and understand their information needs so that the library can respond to these needs, such as through the preparation of recommended titles based on DCT.

Although there are gaps in the collection that need to be filled, the observed temporal trends show that our library attempts to respond to these deficiencies and improve the quality of its collection by acquiring more books based on DCT. It performs regular collection assessments where strong and weak subjects are identified and maps the quality of the book collection. Results of the assessment are considered in acquisitions planning and communicated to school administrators and faculty members to help guide book selection. The library keeps these individuals aware of the relevance of DCT and encourages them to prioritize the selection of books included in DCT. Budget allocated to print collections has increased over time, except in 2020, and funds for the purchase or subscription to electronic books are provided each year, which affords the library more opportunity to acquire DCT-listed titles. Our study demonstrates that title-based acquisition, whether print or online, is effective in keeping the library collection compliant with DCT. Although most subspecialties exhibited an increasing trend in DCT title matching, the associated health professions subspecialty needs particular attention to improve the percentage of matching and halt the decline from 2018 to 2020. Libraries can take advantage of opportunities to build collections for subjects of focus by new programs by becoming more involved in the selection process and building relationships with faculty members to guide them in selection.

DCT is a tool that can be used by medical libraries as a benchmark to assess book collections. However, it is limited to English-language books, may have bias toward the Western practice of health professions, and may not cover the historical and cultural aspects of Asian medicine and allied health education. The availability and cost of DCT-listed titles in Asia could also affect the percentage of matching. However, DCT is helpful in determining the quality of book collections because it covers a range of subspecialties in medicine and allied health sciences that complement the undergraduate and graduate programs offered by the institution. The specializations in nursing and medicine provided by DCT are particularly useful in evaluating the library holdings of undergraduate and graduate programs in nursing and medicine. This makes DCT a relevant tool in collection assessment across all the programs offered and holdings managed by the library. However, this does not imply that other tools for collection assessment are less important. National guidelines and local literature in medicine and allied health sciences should also be considered in developing library collections to cater to the cultural practice and local context.

The library is faced with an opportunity to further develop its collections using DCT in terms of strong and weak areas by maintaining its strong collections, expanding book resources to meet the needs of the new programs, and improving the collections of weak subjects. Title-based selection ensures matching of titles to DCT, whether print or electronic. Electronic books were recently included in the collection assessment, and although they did not contribute much to the match, the library intends to continue its collection assessment using DCT to include both print and electronic books. This opens more opportunities to evaluate the collections by considering formats other than print and to review its acquisitions plans to be able to acquire as many DCT-listed titles as possible. Other collection assessment methods may also be employed in the future to achieve a more holistic view of the state of the collections.
